# Layered Double Hydroxide-Based Nanocarriers for Drug Delivery

**DOI:** 10.3390/pharmaceutics6020298

**Published:** 2014-06-17

**Authors:** Xue Bi, Hui Zhang, Liguang Dou

**Affiliations:** State Key Laboratory of Chemical Resource Engineering, Beijing University of Chemical Technology, P.O. Box 98, Beijing 100029, China; E-Mails: leospindrift@126.com (X.B.); liguang0628@163.com (L.D.)

**Keywords:** layered double hydroxides, drug delivery system, controlled release, intercalation assembly, cardiovascular drug, anti-inflammatory drug, anti-cancer drug

## Abstract

Biocompatible clay materials have attracted particular attention as the efficient drug delivery systems (DDS). In this article, we review developments in the use of layered double hydroxides (LDHs) for controlled drug release and delivery. We show how advances in the ability to synthesize intercalated structures have a significant influence on the development of new applications of these materials. We also show how modification and/or functionalization can lead to new biotechnological and biomedical applications. This review highlights the most recent progresses in research on LDH-based controlled drug delivery systems, focusing mainly on: (i) DDS with cardiovascular drugs as guests; (ii) DDS with anti-inflammatory drugs as guests; and (iii) DDS with anti-cancer drugs as guests. Finally, future prospects for LDH-based drug carriers are also discussed.

## 1. Introduction

### 1.1. Drug Delivery Systems (DDS)

Drug delivery systems (DDS) are designed to either alter the pharmacokinetics and biodistribution of their associated drugs, or to function as drug reservoirs (*i.e.*, as sustained release systems)—or both [1]—and should have the ability to enhance several crucial properties of “free” drugs, such as improving their solubility, *in vivo* stability and specificity, reducing or eliminating tissue damage, protecting the drug, or enhancing their efficacy [2]. Common organic-based DDS include polymers (such as chitosan [3], amphiphilic block copolymers [4], block copolymer micelles [5], hydrogels [6], cellulose [7], polysaccharides [8,9]), lipid particles (microemulsions [10]), and a few natural particulates (pathogens and mammalian) [11]. Organic-based DDS have some disadvantages such as high toxicity, low loadings and easy leakage of drugs which reduce their drug-delivering efficiency. Drug carriers based on inorganic nanomaterials, such as silica materials [12,13], show much better properties than organic carriers, including ease of controlled synthesis and environmental friendliness [14]. The recently developed organic-inorganic nanohybrids based DDS such as LDH–chitosan hybrid [15,16] or enteric polymer [17] show good biocompatibility and avoid the drug leaching, but endure the difficulty of artificial synthesis, ordered structure, and industrial scale-up. Hence, the inorganic materials are much superior to the others on the synthesis and industrialization, the control of defined structure, and the avoidance of drug leakage as DDS in a long-term view. For inorganic materials, efficient administration with low bio-toxicity, facile synthesis, and easy storage with high stability are the most primary factors. The chemical and biological toxicity significantly rely on the choice of chemical elements and the control of particle size. Modification or functionalization by other inorganic or organic components may also create influence on the chemical and biological toxicity of inorganic materials. As for the fast transition of these nanomaterials for industrial scale up, design of assembly technology, data statistics and analysis upon the clinical tests, and long-term storage without loss of pharmacodynamic effect together with the economic evaluation are necessarily required but it is not easy to completely cover in one limited review. The present review mainly focuses on the synthetic strategy and release property *in vitro*. Some layered materials are excellent candidates for use in DDS since their lamellar structures provide a suitable interlayer spacing for drug molecules which can be incorporated, by a process such as ion-exchange [18]. In the case of natural smectite type clays, cationic species may be intercalated in the interlayer galleries, whereas in the case of layered double hydroxides (LDHs)—also known as hydrotalcite-like materials—anionic species may be intercalated. Although some LDHs occur naturally, recent years have seen an explosive growth in the controlled synthesis of new LDH materials and in this review we focus on the prospects of these synthetic LDHs for use in clinical therapy. It is worth mentioning that the pioneering works of Choy’ group have led to a rapid development in the research on both varied LDHs/polymers/anions hybrid systems and pharmaceutical applications of LDHs especially involving the biocompatibility and toxicity of LDHs and anti-cancer drugs intercalated LDH materials.

All these positive attributes make drug–LDH nanocomposite an applicable platform *in vivo* for further evaluation. Drawing together the thoughts and methods from interdisciplinary fields, such as chemistry, biology, mathematics, *etc.*, the drug–LDH materials present great potential in drug imaging or monitoring in human organs or tissues. Therefore, the research scope of this material is no longer limited to conventional studies about structural property and *in vitro* drug release property, but also expands to more subjects like biocompatibility, bio-distribution, or pharmacokinetics, which receive constant attention in clinical work.

### 1.2. Layered Double Hydroxide (LDH) Materials

**Figure 1:**
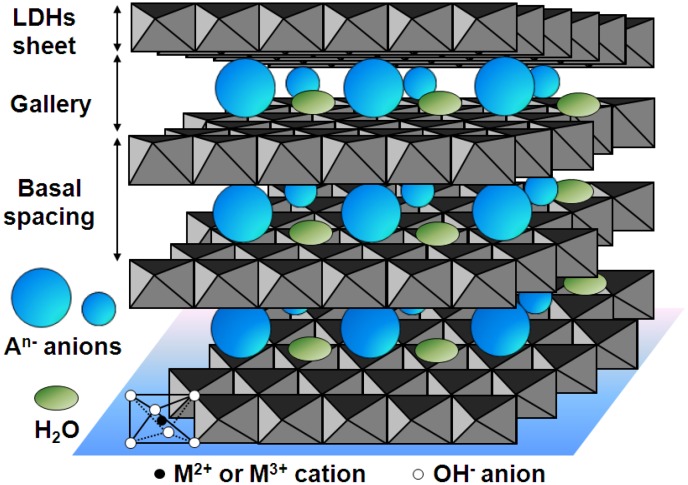
Schematic representation of the structure of layered double hydroxides (LDHs).

LDHs are a class of anionic lamellar compounds made up of positively charged brucite-like layers with an interlayer gallery containing charge compensating anions and water molecules. The metal cations occupy the centers of shared octahedra, whose vertices contain hydroxide ions that connect to form infinite two-dimensional sheets as shown in Figure 1. The most widely studied LDHs contain both divalent and trivalent metal cations and the generic formula for LDHs can be written as: [M^2+^_1–*x*_M^3+^*_x_*(OH)_2_][A^*n*−^]_*x/n*_·*z*H_2_O, where M^2+^ may be cations such as Mg^2+^, Zn^2+^ or Ni^2+^, and M^3+^ may be cations such as Al^3+^, Ga^3+^, Fe^3+^ or Mn^3+^, A*^n^*^−^ is a non-framework charge compensating inorganic or organic anion, e.g., CO_3_^2−^, NO_3_^−^, Cl^−^, SO_4_^2−^, or RCO_2_^−^, and *x* is the mole fraction of M^3+^ [19,20,21,22,23,24]. M^+^ and M^4+^ cations can also be incorporated in the layers but examples are limited to specific cations such as Li^+^, Ti^4+^, and Zr^4+^. In the layers of LDH hosts, the M^2+^ and M^3+^ cations are not randomly distributed but ordered. For example, the solid state NMR studies by Grey *et al.* [25] showed that in MgAl–LDHs the cations are fully ordered for Mg^2+^/Al^3+^ ratios of 2:1 and a nonrandom distribution of the cations, without Al^3+^–Al^3+^ close contacts, persists for higher Mg^2+^/Al^3+^ ratios. The ordering of the cations affects the charge density of the LDH sheets, which has consequences for a variety of physicochemical parameters, such as the bonding, reactivity, orientation, and mobility of the chemical species in the interlayer gallery and on the surface.

LDHs are not only easily synthesized, but also have several other attractive features such as the tunability of layer charge density and particle size, good biocompatibility, low toxicity, and a so-called “structural memory effect”. Based on these properties, LDHs have been widely exploited to create new materials for applications in catalysis [26], drug delivery [27,28], and environmental remediation [29].

### 1.3. LDH-Based Drug Carriers

There are three main attractive features of LDH-based drug carriers: convenient synthesis, structural and morphological tunability, and their low toxicity and good biocompatibility.

#### 1.3.1. Convenient Synthesis

The synthesis of drug–LDH composite materials involves the assembly of organic ions/molecules in the interlayer galleries of the LDH host via electrostatic interactions or hydrogen bonds. O’Hare *et al.*, have reviewed the systematic intercalation chemistry of LDH particles [20] and sheets [23] and highlighted how—in addition to the size of the guest species—the arrangement (monolayer or bilayer), size, and orientation of the guest as well as the interactions between the negatively charged guest and positively charged host are all critical factors in determining the separation between the layers. Several methods are commonly adopted for the synthesis of drug–LDH nanohybrids/nanocomposites, including the coprecipitation, ion-exchange, the calcination-reconstruction, hydrothermal, and exfoliation-restacking methods [17,21,22,23,28,30]. The coprecipitation method is most frequently used for the synthesis of drug–LDH nanohybrids since there is less risk of the incorporation of CO_3_^2−^ or other competing anions [21,28]. The ion-exchange method is also frequently used for the synthesis of drug–LDH nanohybrids [21,30]. LDH materials can also be formed by a calcination-rehydration (or reconstruction) method, in which the mixed metal oxides formed by calcination of an LDH precursor can be rehydrated in the presence of drug molecules to reform the LDH structure with the drug anions intercalated in the interlayer galleries [17]. Some neutral molecules may also be co-intercalated along with anions by this method. The hydrothermal method [24] is mainly used to improve crystallinity and uniformity of LDHs and may be less applicable in the case of drug–LDH nanohybrids if the drug has relatively low thermal stability. Although LDHs are harder to exfoliate than smectite clays because of their high layer charge density, several ways of achieving exfoliated LDHs have been developed in recent years and the restacking of the exfoliated nanosheets in the presence of drug species offers an attractive way of synthesizing drug–LDH nanohybrids under mild conditions, which is worthy of further exploration [23].

It should also be noted that the properties of drug–LDH nanohybrids/nanocomposites can be extended by incorporating a third functional component, such as organic [31,32,33,34,35], SiO_2_ [36,37], and Fe_3_O_4_ [38,39,40].

**Figure 2:**
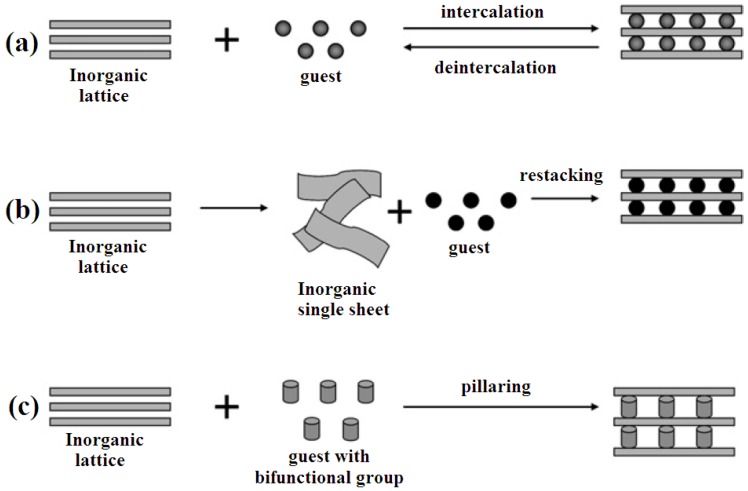
Reaction routes to incorporate biomolecules into layered nanomaterials (**a**) intercalation; (**b**) exfoliation-restacking; (**c**) pillaring reaction. Reprinted with permission from [41] (Copyright 2009 Royal Society of Chemistry).

Some of the reaction routes to incorporate biomolecules into layered nanomaterials are summarized in Figure 2 [41]. For a specific drug–LDH composite, the choice of method is determined by a variety of factors such as the requirements on particle size, crystallinity, and loading amount of the drug. As it is shown in Figure 2, the inorganic sheets of a layered material such as an LDH act as a protective shield and their two-dimensional interlayer provide an ordered spacing which can effectively arrange the guest molecules.

#### 1.3.2. Structural and Morphological Tunability

The availability of LDH hosts with a wide range of chemical compositions allows materials with different structures and morphologies to be fabricated. For example, Duan *et al.* [30] showed that 5-aminosalicylate (5-ASA) intercalated ZnAl–LDH via direct coprecipitation and indirect ion-exchange methods with different Zn^2+^/Al^3+^ molar ratios had a variety of distinct arrangements of the 5-ASA guests in the interlayer galleries with various gallery heights.

Although the crystallites of LDH materials generally have a hexagonal plate-like morphology, the particle size can be readily controlled according to the demands of a specific application. For example, Xu *et al.* [42] fabricated MgAl–LDH hollow nanospheres via direct assembly of preformed anisotropic LDH nanocrystals on the surface of carbon nanospheres followed calcinations and reconstruction to obtain ibuprofen (IBU)-intercalated LDH with ~400 nm hollow nanosphere morphology. Zhang *et al.* [43] synthesized IBU-intercalated MgAl and ZnAl–LDH nanohybrids by the coprecipitation followed hydrothermal treatment and found that the average plate-like particle sizes of the IBU–LDH nanohybrids can be tuned between 350 and 530 nm by modulating the aging time.

#### 1.3.3. Low Toxicity and Good Biocompatibility

Many of the divalent and trivalent metal cations which can form LDHs have low toxicity. MgAl–LDHs are most frequently used as a LDH-based drug carrier and a significant number of studies have also been carried out with ZnAl–LDHs. As evidence of its low toxicity, MgAl–LDH–CO_3_ is widely used as an antacid [44]. LDH hosts have the advantage that many different guest anions—including not only drugs, but also important agrochemicals, vitamins, fragrances, and dyes—can be intercalated without any degradation or loss of structural integrity of the guest, allowing the LDHs to act as a reservoir of the guest species [45]. Moreover, in addition to the intercalation of pharmaceutical drugs into layered materials causing no significant denaturation of the drug molecules, it has also been shown to enhance the internalization of the drug into a target cell without any noticeable side effects [41]. For example, 5-fluorouracil (5-Fu)–LDH exhibits favorable blood clearance profiles compared to free 5-Fu, such as sustained drug release, prolonged drug half-life, and increased drug accumulation in target tumor tissue [46]. Furthermore, LDH nanoparticles are rapidly excreted from the body and not accumulated in the organs after administration as 5-Fu–LDH, showing their efficacy as biodegradable particles for drug delivery [47]. Similarly, the greater inhibition of the cell cycle by methotrexate (MTX)–LDH compared with pure MTX has been demonstrated [48], and it was proposed that the internalization of LDH nanoparticles via clathrin-mediated endocytosis may allow the efficient delivery of MTX–LDH into cells and thus enhance drug efficiency.

Thus, LDHs can not only play a role as a biocompatible-delivery matrix for drugs but also afford a significant increase in the delivery efficiency [49,50,51,52,53]. However, before actual applications can be considered, the toxicity of drug–LDH composites in terms of damage to human tissue and organs must be studied. Choy *et al.* compared the toxicological effects of different nanoparticles focusing on their cytotoxicity and toxicity *in vivo* [51]. Choy *et al.* [52,53] further deduced that although LDH nanoparticles exhibit some cytotoxic effects, they are less toxic than other inorganic nanoparticles such as iron oxide, silica, and single walled carbon nanotubes at practical concentration levels. Although LDHs have low toxicity, high concentrations may still cause adverse effects [47]. Many toxicological studies of nanoparticles have demonstrated that the most important factor in determining the toxicity is the size of the particles themselves because they generally show size-dependent toxicity [54]. Examination of the size-dependent toxicity of LDHs in human lung cell cultures showed that 50 nm particles are more toxic than larger particles, while LDHs within the size range of 100–200 nm exhibits very low cytotoxicity in terms of cell proliferation, membrane damage, and inflammation response [55]. Besides particle size, the stability of an LDH also depends on the type of interlayer anions, and this can also affect the toxicity. It was reported [56] that MgAl–LDH–Cl dissolved more readily under simulated lysosomal (pH 4.5) and body fluid (pH 7.4) conditions than MgAl–LDH–CO_3_, and exhibited lower toxicity in terms of induction of oxidative stress, apoptosis and membrane damage.

## 2. Drug–LDH DDS

### 2.1. Cardiovascular Drugs as Guests

According to WHO (the World Health Organization), cardiovascular diseases rank first in the cause of death globally leading to strong demand for cardiovascular drugs with durable efficacy. To date, several cardiovascular drugs have been successfully integrated into LDH hosts for controlled drug delivery, indicating that LDHs are potential nanocarriers for the treatment of cardiovascular diseases.

**Figure 3:**
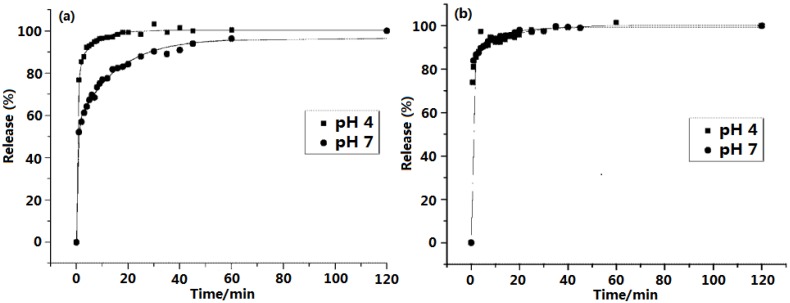
Release profiles for (**a**) diclofenac at pH 4 and pH 7 and (**b**) gemfibrozil at pH 4 and pH 7. Reprinted with permission from [57] (Copyright 2001 Royal Society of Chemistry).

As early as 2001, O’Hare *et al.* [57] reported the reversible intercalation of a number of active cardiovascular and anti-inflammatory agents into LDHs. As shown in Figure 3, at pH 4 the measured release of gemfibrozil is very fast with almost full deintercalation in less than 10 min. While the release curve for gemfibrozil at pH 7 is almost identical to the profile at pH 4. These results show that the intercalation of pharmaceutically active compounds that can form stable anions is a feasible approach for the storage, and subsequent controlled release, of bioactive agents.

**Figure 4:**
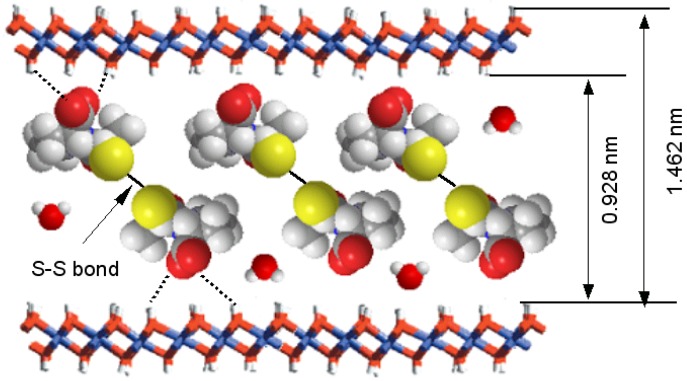
Supramolecular structural model of Cpl–LDH. Reprinted with permission from [58] (Copyright 2006 Elsevier).

In 2006, Zhang *et al.* [58] developed a nanostructured drug–LDH composite involving a pharmaceutically active cardiovascular drug captopril (Cpl) intercalated in an Mg_2_Al–LDH host by a coprecipitation method. The interlayer spacing of the Cpl–LDH, evaluated by subtracting LDH layer thickness 0.48 nm from the interlayer distance *d*_003_ (measured by powder X-ray diffraction (XRD)), is 0.982 nm, which is larger than the molecular size of Cpl (0.526 nm) and this, together with Fourier transform infrared (FT-IR) and Raman spectroscopy data, suggest a vertical arrangement of a Cpl disulphide containing an S–S bond in interlayer gallery, with carboxylate anions interacting with both metal hydroxide layers. The proposed supramolecular structure of Cpl–LDH is presented in Figure 4.

**Figure 5:**
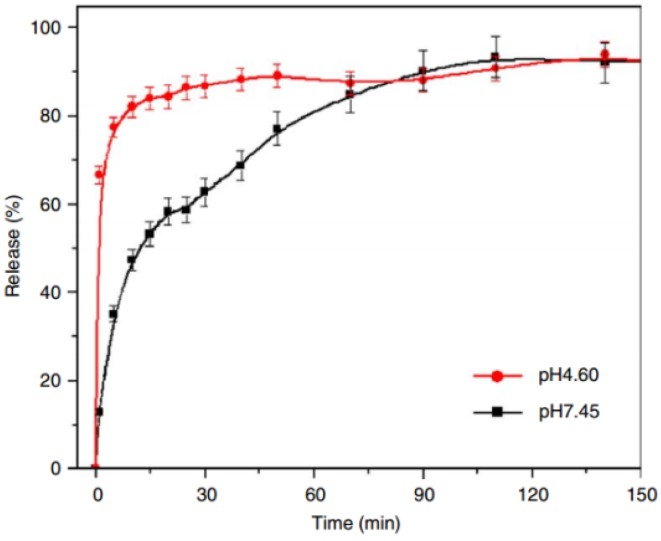
Release profiles of Cpl from Cpl–LDH in buffer solutions at different pH values Reprinted with permission from [58] (Copyright 2006 Elsevier).

The *in vitro* release studies of Cpl–LDH in simulated gastrointestinal solutions at 37 ± 1 °C (Figure 5) showed that at pH 4.60, followed initial fast release, a slower release step is characterized by percentage releases of *ca.* 66.7%, 85.1% and 94.2% after 1, 10 and 140 min, respectively, while at pH 7.45, a slow and persistent process of *ca.* 12.8%, 47.4% and 92.4% are obtained after 1, 10 and 140 min, respectively. It is worth noticing that no burst release phenomenon occurred in pH 7.45 solution. Kinetic simulation of the release data and characterization results for samples recovered after release test indicate that a dissolution mechanism is mainly responsible for the release behavior of Cpl–LDH at pH 4.60, while an ion-exchange mechanism for that at pH 7.45.

**Figure 6:**
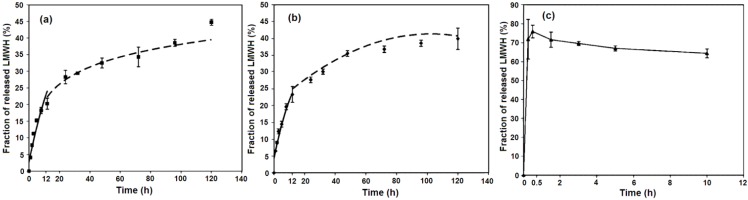
*In vitro* Low molecular weight heparin (LMWH) release curves from (**a**) LMWH20–LDH; (**b**) LMWH100–LDH; (**c**) physically mixed powder of heparin sodium salt and Cl–LDH (1/8 weight ratio). In (**a**) and (**b**), the solid and dashed curves represent the predictions of the modified Freundlich and parabolic diffusion model, respectively. Reprinted with permission from [59] (Copyright 2008 American Chemical Society).

Low molecular weight heparin (LMWH) has also been intercalated into LDH hosts. In 2008, Gu *et al.* [59] prepared Mg_2.4_Al*–*LDH–LMWH hybrids with the desired LMWH loading (LMWH*_n_*–LDH, *n* = 10, 20, 50, or 100, where *n* denotes the percentage of Cl^−^ ions in pristine Mg_2.4_Al*–*LDH–Cl that are replaced by LMWH anions) using a similar coprecipitation method, aiming at overcoming the pharmaceutical limitations of heparin, namely, short half-life, low efficiency of cellular delivery, and lack of oral absorption. The *in vitro* release profiles in pH 7.4 phosphate buffered saline (PBS) (Figure 6) show that LMWH20–LDH exhibits a gradual and biphasic release pattern, with an early fast release of LMWH (20.3% of LMWH released after the first 12 h) followed by a relatively slower release (44.7% released after 120 h), while LMWH100–LDH shows a similar biphasic release pattern, in which 23.3% of LMWH was released in the first 12 h and 39.9% in 120 h. The mechanism of release of LMWH from the LDH host probably involves surface diffusion and bulk diffusion via anionic exchange of LMWH anions on, or in, the LDHs with anions in the PBS solution.

**Figure 7:**
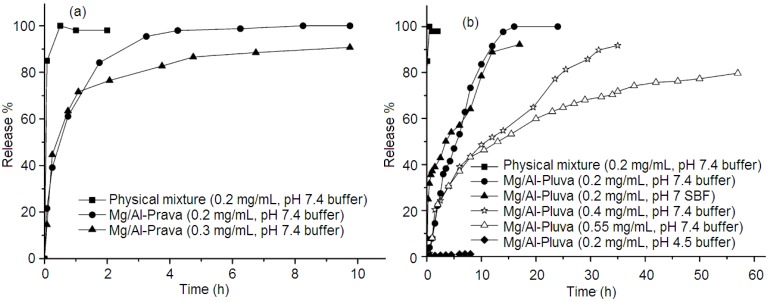
Release profile of statin drugs for (**a**) pravastatin and (**b**) fluvastatin based LDH systems under various physiological conditions. Reprinted with permission from [60] (Copyright 2009 American Chemical Society).

Pravastatin (prava) and fluvastatin (fluva) are members of the statin family of drugs that are highly effective for reducing cholesterol levels in the blood stream. In 2009, Panda *et al.* [60] reported the intercalation of pravastatin and fluvastatin drugs into MgAl*–*LDHs via the coprecipitation technique. Characterization and computational results indicated that the fluvastatin anions are attached with the brucite as a hydrophobic monolayer, while the pravastatin anions form a hydrophilic multilayer. *In vitro* release study (Figure 7) indicated that although both the drugs contain single charged anions and the drug–LDH nanohybrids show a monophasic release pattern at pH 7.4 as reported earlier for other drugs, MgAl*–*LDH–fluva exhibits a gradual and slow release pattern. In both cases, there is an early fast release (for pravastatin of 40% and fluvastatin of 20%) of drug ions followed by a relatively slower release for fluvastatin. Also, on varying the concentration of hybrids materials in solution, there is a significant change in release patterns for both MgAl*–*LDH–prava and MgAl*–*LDH–fluva hybrids. For MgAl*–*LDH–fluva, a concentration of 0.2 mg·mL^−1^ gave 100% release in 16 h, a concentration of 0.4 mg·mL^−1^ gave 85% release in 32 h whilst a concentration of 0.55 mg·mL^−1^ gave only 79% release in 52 h; for MgAl*–*LDH–prava, a concentration of 0.2 mg·mL^−1^ gave 100% release in 10 h and a concentration of 0.3 mg·mL^−1^ gave 91% release in 10 h. The great reduction in release rate of fluvastatin ions from MgAl*–*LDH–fluva upon materials concentration is ascribed to its hydrophobic nature, and this provides further means of controlling the rate of release in a physiological medium. The mechanism of drug diffusion in the hydrophobic MgAl*–*LDH–fluva nanohybrid probably involves heterogeneous diffusion via anion-exchange, while in the hydrophilic MgAl*–*LDH–prava nanohybrid it is due to intraparticle diffusion via anion-exchange with the anions in the physiological medium.

Fibrates such as bezafibrate (BZF) and clofibric acid (CF) are a class of lipid-regulating drugs that have been used in the treatment of many forms of hyperlipoproteinemia. In 2010, Berber *et al.* [61] used the coprecipitation method to intercalate BFZ and CF into an MgAl*–*LDH host; drug loadings of 54% and 45%, respectively, were reached. The controlled release properties of BZF–LDH and CF–LDH were attributed to the presence of anionic species in simulated gastrointestinal solutions. From the release data of BZF–LDH and CF–LDH, the authors concluded that the nanocomposite formulation with LDHs facilitated the drug release properties.

### 2.2. Anti-Inflammatory Drugs as Guests

Non-steroidal anti-inflammatory drugs (NSAIDs) are aromatic organic compounds with easily ionizable carboxylic groups, thus permitting their intercalation as anions between the layers of LDH hosts [62]. It has been shown that many common NSAIDs, such as ibuprofen [42,43,63,64,65,66,67,68], naproxen [69,70,71,72,73,74], diclofenac [75,76,77,78], and some other drugs [30,79,80,81,82,83,84] can be rapidly intercalated in LDH hosts using a variety of methods, mainly coprecipitation, ion-exchange, and reconstruction.

Ibuprofen is a prototypical NSAID with analgesic and antipyretic properties and has been most widely selected as the guest. In 2001, Grandolini *et al.* [63] reported the intercalation of IBU in LDH–Cl via ion-exchange method. After intercalation of IBU, the interlayer distance of the host increased from 0.78 nm (parent LDH–Cl) to 2.17 nm. The dissolution tests in simulated intestinal fluid buffer (PBS, pH 7.5) showed 60% of drug release at 20 min and 100% at 100 min, quite different from those of physical mixture and commercial formulation Neo-Mindol^®^. The mechanism of modified drug release was interpreted on the ion-exchange of the interlayer IBU anions with phosphate anions in PBS.

Subsequently, Silva *et al.* [64] reported the immobilization of ibuprofen on Mg_3_Al*–*LDH by three routes compared with that of the Cu(II)–ibuprofen salt by adsorption method. Evaluation of buffering properties showed that Mg_3_Al*–*(ibuprofen) by the reconstruction method combines a significant amount (13%) of immobilized ibuprofen with good buffering properties whilst the Mg_3_Al*–*(ibuprofen) samples by ion-exchange and coprecipitation methods could be good carriers for the drug due to their higher ibuprofen loading (~33%) despite their poor buffering properties.

**Figure 8:**
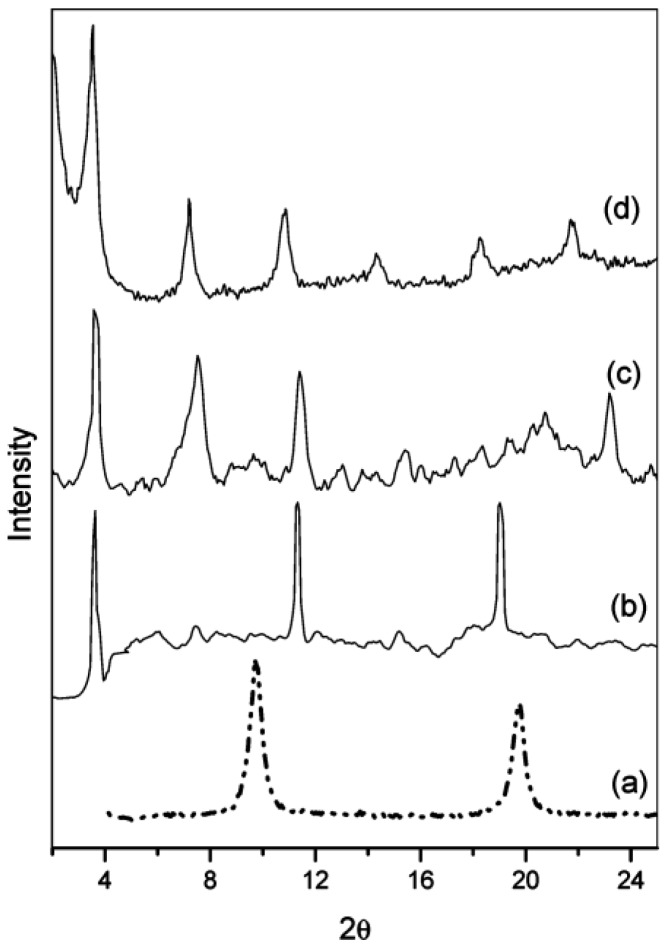
X-ray diffraction patterns of (a) MgAl*–*LDH–NO_3_; (b) MgAl*–*LDH–ibuprofen; (c) MgAl*–*LDH–diclofenac; and (d) MgAl*–*LDH–indomethacin. Reprinted with permission from [65] (Copyright 2005 American Chemical Society).

Three representative NSAIDs, ibuprofen, diclofenac, and indomethacin, were intercalated within the galleries of MgAl*–*LDHs via ion-exchange step by Vasudevan *et al.* [65]. The XRD patterns (Figure 8) showed that the (003) diffractions of precursor MgAl*–*LDH*–*NO_3_ disappear instead of those of drug–LDH hybrids at lower 2θ angles, indicating the completeness of the ion-exchange processes. The gallery height of 1.88 nm for MgAl*–*LDH–ibuprofen may be accounted for a bilayer arrangement with the carboxylate group of the ibuprofen anion (length 0.96 nm) anchored to the hydroxide layer. Given that the dimensions and geometry of the diclofenac and indomethacin molecules are the same as in their crystalline state, the authors believed that the observed gallery heights 1.81 and 1.97 nm for MgAl*–*LDH–diclofenac and MgAl*–*LDH–indomethacin, respectively, implied partially interdigitated bilayers of the drug molecules arranged between the LDH layers. These hybrid materials have been identified as potential candidates for pH-triggered drug release as well as drug storage.

**Figure 9:**
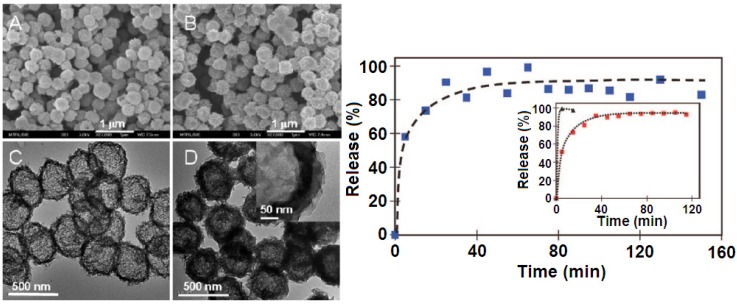
(**Left**) Scanning electron microscopy (SEM) (**A**) and transmission electron microscopy (TEM) (**C**) of metal oxide hollow nanospheres obtained after calcinations of LDH*-*NCs (nanocrystals)/central nervous system (CNS) composite and SEM (**B**) and TEM (**D**) of ibuprofen (IBU)*-*LDH hollow nanospheres after reconstruction; (**Right**) *In vitro* release profile of IBU from IBU-LDH hollow nanospheres in a buffer solution pH 7.0. The inset shows the release profiles from IBU–LDH nanoplates (■) and IBU sodium salt (▲) under the same conditions. Modified with permission from [42] (Copyright 2009 American Chemical Society).

In 2008, Xu *et al.* [66] reported the particles interactions-dependent control of drug release from Mg_2_Al–LDHs intercalated with IBU through coprecipitation coupled with atmospheric aging or hydrothermal treatment in varied solvent. The sample obtained in ethylene glycol(EG)/water (volume ratio of 1:1) under hydrothermal condition forms c-oriented dense powder due to the preferential surface-to-surface and edge-to-edge aggregations of the large, regularly shaped and crystalline drug*–*LDH platelets. As a result, the release rate of IBU was considerably slower from the dense and oriented sample than that from loosely aggregated powders, due to the longer average diffusion path and higher diffusion resistance. These authors also demonstrated a simple method for generating LDH hollow nanospheres (Figure 9) via direct assembly of preformed anisotropic LDH nanocrystals (NCs) on the surface of carbon nanospheres [42]. The *in vitro* release study showed that there is no substantial difference in the release profiles between IBU–LDH hollow nanospheres and IBU–LDH nanoplates. However, the former has several advantages, such as (i) a lower density and less aggregation compared to the nanoplates, which leads to a better dispersion in the liquid phase for potential applications in controlled drug release with an intravenous injection mode; (ii) a higher surface area (53.9 m^2^·g^−1^ compared to 14.7 m^2^·g^−1^ for LDH nanoplates) allowing more effective surface modification by functional species (e.g., polymers or silica); and (iii) the interior space of the hollow nanospheres can be used for encapsulation of other molecules or nanoparticles (e.g., dyes or magnetic nanoparticles) to make multifunctional nanocomposites.

**Figure 10:**
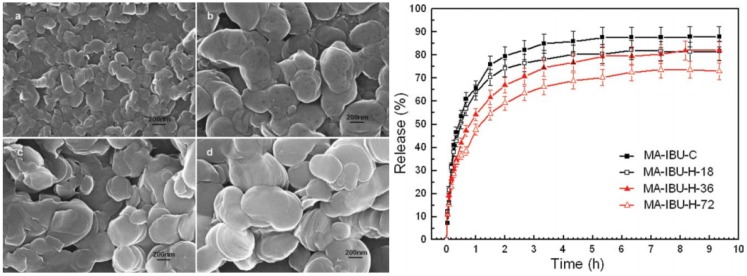
(**Left**) SEM images of MA-IBU*-*C (**a**) and MA-IBU*-*H*-i* samples with *i* = 18 h (**b**), 36 h (**c**) and 72 h (**d**); (**Right**) Release profiles of IBU from MA*-*IBU*-*C (■), MA*-*IBU*-*H*-*18 (□), MA*-*IBU*-*H*-*36 (▲) and MA-IBU-H-72 (∆) in pH 7.45 PBS. Modified with permission from [43] (Copyright 2010 American Institute of Chemical Engineers).

In 2011, Zhang *et al.* [43] fabricated MgAl*–*LDH nanohybrids intercalated with IBU through hydrothermal (H) and coprecipitation (C) methods in aqueous solution without any organic solvent. The (001) diffractions of the MA-IBU*-*H samples are obviously sharper than those of MA-IBU*-*C, indicating that the hydrothermal treatment leads to higher product crystallinity. The crystallinities and particle sizes of the MA*-*IBU*-*H*-i* (*i* = 18, 36, 72 h) hybrids are greatly improved with increasing aging time indicated by the progressive decrease in the peak width of the (110) peak. The SEM (Figure 10) and TEM images show typical sheet-like morphology of all intercalates, with the platelet particles stacked and mostly adhering to one another. The average particle sizes of MA*-*IBU*-*H*-i* nanohybrids are increased gradually from 350 to 530 nm with increasing aging time, and significantly larger than that of MA*-*IBU-C (~150 nm). *In vitro* release studies for all the four intercalates in pH 7.45 PBS indicated that the particle size has an important effect on the release rate and equilibrium. By quasi-in-time monitoring of the morphological changes of large-sized drug–LDH particles during the release process, it was found that the stacked and adhered platelet drug–LDH nanoparticles were gradually converted into isolated and thin plate-like LDH nanoparticles with curved edges.

D’Souza and Golden [67] reported the intercalation of IBU in inorganic biodegradable polymer composites of LDH and poly-l-lactic acid (PLLA). The basal spacing expands from 0.89 to 2.48 nm (3% IBU*–*LDH loaded) and 2.66 nm (5% IBU*–*LDH loaded) with a titled double-layer arrangement of IBU within the gallery. Cumulative drug release was significantly improved by virtue of the novel nanocomposite architecture. Drug release studies showed a two-stage mechanism for release of the IBU from the nanocomposite films, but a single stage release from non-nano analogues. It was shown that the LDH works synergistically to strengthen PLLA while facilitating drug release. At shorter times periods (<5 h), the drug was released by diffusion, while ion-exchange predominates at longer time periods. These studies showed that ibuprofen could be intercalated into LDHs by an ion-exchange mechanism, maximizing therapeutic activity while minimizing toxic side effects.

Duan *et al.* [68] investigated the thermal decomposition process of naproxen-intercalated MgAl*–*LDHs by *in situ* FT-IR, *in situ* variable temperature XRD and thermogravimetry. It was found that the thermal stability of the intercalated naproxen was significantly enhanced compared with the pure form before intercalation, which suggests that this drug-inorganic layered material may have potential application as the basis of a novel drug storage system.

Berber *et al.* [69] reported the intercalation of naproxen in a MgAl*–*LDH by coprecipitation and studied the effect of the inorganic matrix on the drug solubility at pH 2. The LDH dissolved in the acidic medium and the intercalated drug was released in a molecular form which is suitable for absorption. The solubility of the drug after intercalation was compared with that of the free drug. It was found that intercalation in the LDH host improves the drug solubility and its dissolution rate.

Rives *et al.* [70] have also studied the intercalation of naproxen in LDHs by both coprecipitation and anion-exchange with the Al^3+^ cations in the brucite layers partially substituted by Fe^3+^. In addition, the dissolution rate of the drugs was studied *in vitro*, in order to ascertain whether the LDH acts as a simple additive or as a matrix. The drug species, in their anionic form, were intercalated forming bilayers and during dissolution were exchanged with anions in the medium. This led to a slow release, much slower than when the LDH was simply and physically mixed with the drug, or when MgAl*–*LDH matrixes were used. These workers also reported the synthesis and characterization of materials where naproxen was incorporated in different inorganic matrices (MgAl–LDH, MCM-41 mesoporous silica and mesoporous alumina) [71], and the sustained release of the drug was compared using *in vitro* experiments. For the naproxen–LDH material, the amount of naproxen incorporated is much higher than for the mesoporous systems, as the drug molecules are located in the interlayers forming a bilayer to balancing the positive charge of the brucite-like layers.

Hou and Jin [72] reported the intercalation of naproxen into ZnAl*–*LDHs by the ion-exchange method involving the effects of varying the contact time, the composition, the layer charge density and the specific surface area of the LDHs, as well as the pH value on the uptake and release of naproxen by the LDHs. In the pH range of 6–11.5, the amount of naproxen uptaken by LDHs reduces with increasing pH values. The uptake by LDH–Cl is much higher than that by LDH–CO_3_ being probably due to the higher anion-exchange ability of the former. The naproxen molecules are possibly adsorbed on the surface of the basal layer of the LDH. In other words, a bilayer is formed in the interlayer gallery of the LDH host. They also found that the release rate of naproxen from the naproxen–LDH decreases with increasing charge density on the LDH layers and is much lower than that of a naproxen troche, implying an efficient drug-controlled release system of the naproxen–LDH nanohybrid.

Ricci *et al.* [73] prepared a microencapsulation of an MgAl*–*LDH interaclated with diclofenac (HTlc*–*DIK) in order to obtain a composite system suitable for colonic drug delivery. Eudragit^®^ S or Eudragit^®^ L were used as polymers for preparation of the microparticles (MPs) with two different HTlc-DIK/polymer ratios. DIK release from Eudragit^®^ L MPs at pH 6.8 reached 26%–35% within 25–30 min for the different HTlc*–*DIK/polymer ratios and no further increase was observed when the pH was increased to 7.5. DIK release from Eudragit^®^ S MPs at pH 7.5 reached 70% after 6–8 h for both HTlc*–*DIK/polymer ratios. The results showed that LDH can be microencapsulated without loss of structural integrity and the resulting composite MPs showed good release property. Overall HTlc*-*DIK/Eudragit^®^ S MPs possess superior features with respect to HTlc*–*DIK/Eudragit^®^ L MPs suggesting that they could be promising systems for drug delivery either to the distal part of the small intestine or to the colon.

**Figure 11:**
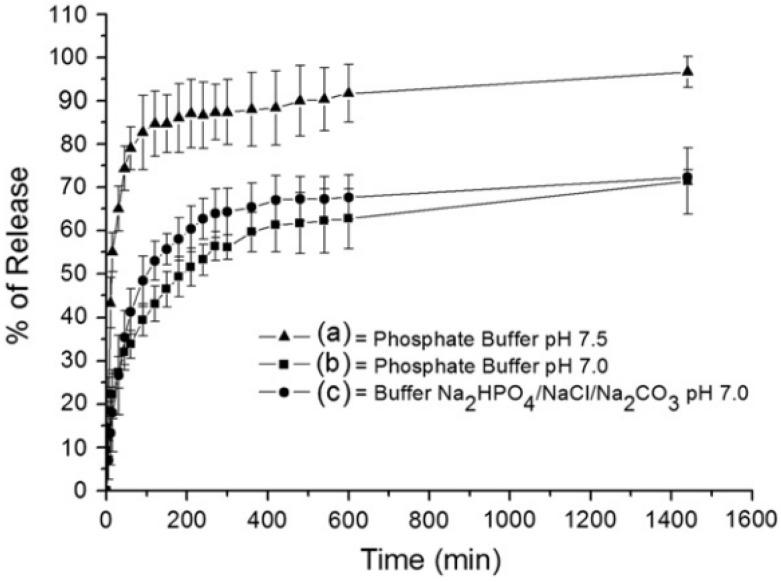
Release curves of DIK from ZnAl–LDH–DIK under different conditions. Reprinted with permission from [74] (Copyright 2011 Elsevier).

Cipiciani *et al.* [74] selected a [Zn_0.72_Al_0.28_(OH)_2_]Br_0.28_·0.69H_2_O LDH as the host of the anionic to form diclofenac–LDH by ion-exchange method. The drug release profiles at different pH and in different media (Figure 11) showed that the drug release was complete in phosphate buffer at pH 7.5 but incomplete in pH 7.0 phosphate and pH 7.0 Na_2_HPO_4_/NaCl/Na_2_CO_3_ buffers, suggesting that the rate of release is more sensitive to acidic H_2_PO_4_^−^ ions than to CO_3_^2−^ or Cl^−^ ions.

Rives *et al.* [75] also studied the intercalation of diclofenac, chloramphenicol, and ketoprofen into ZnAl, MgAl, ZnMgAl–LDH by coprecipitation method. The resulting nanohybrids show a relatively high crystallinity with drug molecules interacting strongly with the brucite-like layers. They also [76] attempted to modify the controlled release characteristics by incorporating the drug–LDHs into the biodegradable polymer PLLA and found that the release rate of drug was dramatically reduced and was not complete even after 3 months for any of the samples. Only 36% of ketoprofen was released from the drug–LDH–PLLA system after three months, while the amounts released in the cases of diclofenac and chloramphenicol were 24% and 70%, respectively. They pointed out that nevertheless, the net amounts of drug released might be therapeutically appropriated in all cases, as the amount of drug reaching the tissue would be of the same order as that obtained by oral dosing and the presented release pathway might be also beneficial for the interaction between the drug and the tissue.

About other anti-inflammatory drugs, Duan *et al.* [77] reported the intercalation of the fenbufen into LDHs by coprecipitation route associated with intercalation conditions such as pH value of the solution and chemical composition of the host. The interlayer distance in the intercalated materials increased with increasing pH value, resulting from a change in the arrangement of interlayer anions from monolayer to interdigitated bilayer. Drug release characteristics of the pillared LDH materials were investigated by a dissolution test in a simulated intestinal fluid (pH 7.8). The results showed that the release of the drug from the supramolecular LDH materials is a slow process, especially in the case of Mg/Al intercalated materials, suggesting that these drug-inorganic hybrids can be used as an effective drug delivery system. They subsequently prepared a core-shell material [78] using fenbufen–LDH as the core coated with enteric polymers, Eudragit^®^ S 100 or Eudragit^®^ L 100 as a shell, giving a composite with controlled drug release behavior under *in vitro* simulated gastrointestinal tract conditions.

Rives *et al.* [79] also studied the intercalation of fenbufen into LDHs containing Mg^2+^ and Al^3+^ or Mg^2+^, Al^3+^ and Fe^3+^ cations by reconstruction route, with interlayer spacings ranging between 1.61 and 1.88 nm. The presence of the LDH increases the s the drug is olubility of fenbufen, especially when used as a matrix. The dissolution rate of the drug decreases when intercalated, and is even lower in those systems containing iron; release takes place through ion-exchange with phosphate anions in the solution. Preparation of microspheres with Eudragit^®^ S 100 afforded solids with a homogeneous, smooth surface with efficient covering of the LDH surface, as drug release was not observed at pH lower than 7, showing that the Eudragit^®^ S 100 acts as an effective enteric coating for drug–LDH.

Another NSAID flurbiprofen (FLUR), belonging to class II of the Biopharmaceutical Classification System (BCS), was intercalated into LDHs by Perioli *et al.* [80] in order to evaluate the effect of the intercalation on the drug dissolution rate in an acid medium, the FLUR gastric permeability in the presence of MgAl–LDHs, and the *in vitro* FLUR release profile in an intestinal environment. It was found that the lamellar structure of LDHs quickly dissolves releasing the drug in a molecular form in acidic conditions; the co-administration of MgAl–LDH ensures not only an antacid effect but also affords an improvement in drug permeation through the gastric mucus which would probably lead to a better drug absorption *in vivo*. Choy *et al.* [81] also reported the intercalation of FLUR into LDH via a coprecipitation step. They showed that the nanohybrids can release the drug in a sustained manner, which can be better controlled with the presence of a macromolecule with comparable polarity to the incorporated drug. Upon the *in vitro* release results, the drug release could be facilitated by the presence of an anionic macromolecule, Eudragit^®^ S 100, in the release medium.

5-Aminosalicylate, widely used in the treatment of inflammatory bowel disease, including ulcerative colitis and Crohn’s disease, was intercalated into Zn*_R_*Al–LDH (*R* is Zn/Al molar ratio) by Duan *et al.* [30] through both direct coprecipitation (CP) and indirect ion-exchange (EX) methods. It was found that the EX products possess more ordered crystallites than the CP ones and the intercalates with higher layer charge density, *i.e.*, lower Zn/Al ratio, and exhibit more ordered crystal structures and higher thermal stability than those with lower layer charge densities. For EX intercalates, when the available area per monovalent anion is smaller than the cross section of the 5-aminosalicylate ion (ASA^−^), the interlayer ASA^−^ ions adopt a staggered interpenetrating arrangement, whereas when the available area is sufficiently large, the ASA^−^ ions adopt the vertical neighboring monolayer arrangement. For CP intercalates, when the available area per monovalent anion is smaller than the cross section of the ASA^−^ ion, the interlayer ASA^−^ ions adopt a vertically distant bilayer arrangement, whereas when the available area is sufficiently large the ASA^−^ ions adopt a vertical neighboring bilayer arrangement.

Indomethacin was intercalated into Mg_2_Al–LDHs by Rives *et al.* [82] through calcinations-reconstruction and coprecipitation routes. The coprecipitation sample shows a single layered structure while the reconstruction sample with contamination of another layered MgAl–CO_3_ phase and the amount of drug intercalated, as well as the height of the gallery, is larger for the former. Pharmacological studies *in vivo* showed that intercalation of the drug in the LDH host reduced the ulcerating damage of the drug. The same group also reported the intercalation of mefenamic and meclofenamic acid anions in Mg_2_Al–LDHs by coprecipitation, ion-exchange and reconstruction methods [83]. Intercalation was achieved using all three methods, with the gallery height ranging between 1.65 and 1.75 nm depending on the particular drying conditions; these values suggested that the organic molecules form bilayers in the interlayer space.

A dexamethasone (Dexa) sodium phosphate was intercalated into MgAl–LDHs by Bahadur *et al.* [84] using the coprecipitation technique. An *in vitro*release study of the nanohybrid particles showed a significant reduction in release rate of dexamethasone phosphate anions from MgAl–LDH–Dexa after confinement of the drug anions in the LDH interlayer gallery. The mechanism of drug diffusion in the nanohybrid was studied using the dissolution-diffusion kinetic model, and it was found that it is probably due to dissolution and intraparticle diffusion of anions in the physiological medium.

### 2.3. Anti-Cancer Drug as Guests

As a disease with high mortality and few effective therapies, cancer is an international problem within the medical profession. Riaz and Ashaf [85] have given a short review of recent research into the intercalation and delivery of anti-cancer drugs using LDHs. To date, LDHs have been utilized as nanocarriers for many anti-cancer drugs, such as folinic acid [48], methotrexate [48,50,86,87,88,89,90,91,92,93], 5-flourouracil [94,95,96,97] and doxifluridine [17], comptothecin [98,99,100], K_7_[PTiW_10_O_40_]·6H_2_O [101], podophyllotoxin [102], and doxorubicin [103].

**Figure 12:**
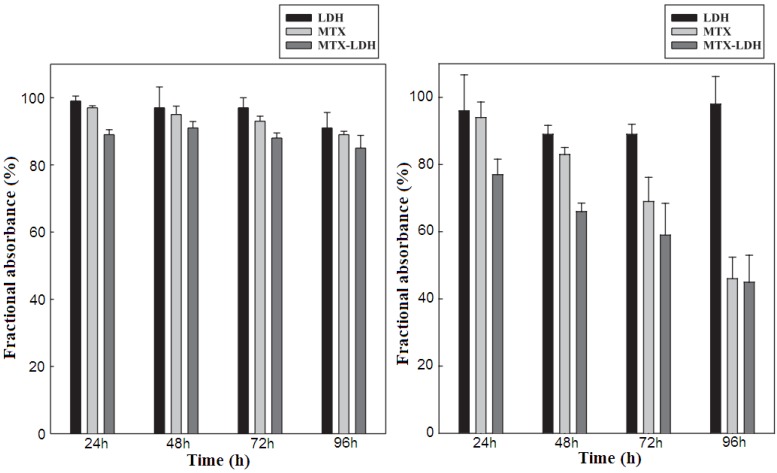
The effect of methotrexate MTX–LDH on normal (**Left**) and tumor (**Right**) cell growth at the concentration of 5.0 μg·mL^−1^. Reprinted with permission from [48] (Copyright 2004 Elsevier).

Methotrexate (MTX), a folic acid antagonist commonly used to treat cancer sufferers, but with very short plasma half-life, leads to administering a high dose that can cause drug resistance and nonspecific toxicities in normal proliferating cells and therefore needs to be modified by organic or inorganic nanoparticles. About the modification of MTX by LDH, Choy *et al.* made significant contributions [48]. Early in 2004, Choy *et al.* [48] prepared MTX–MgAl–LDH hybrid via ion-exchange method from Mg_2_Al–NO_3_ precursor. *In vitro* bioassay (Figure 12) showed that during the initial period, MTX–LDH is more efficient than MTX in suppression of the proliferation of tumor cell (fibroblast (human tendon) and SaOS-2 (Osteosarcoma, human)), and the clear difference in drug efficiency between MTX–LDH and MTX lasts for three days after administration. This result indicated that delivery to tumor cells is noticeably enhanced by hybridization with LDH and in this system MTX can reach the tumor cell membrane without any early decomposition due to stabilization and protection from the LDH host.

**Figure 13:**
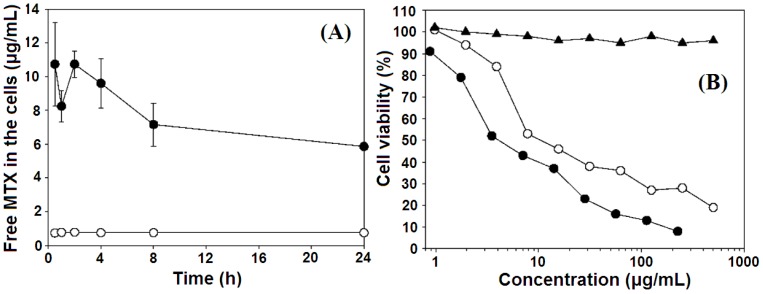
Cellular accumulation of free MTX molecules in MNNG/HOS cells treated with either MTX (○) or MTX–LDH (●). (**A**) Cell viability/cytotoxicity of MNNG/HOS cells treated with LDHs (▲), MTX (○), and MTX–LDH (●), as monitored by trypan blue exclusion, with respect to drug concentration (**B**). Reprinted with permission from [50] (Copyright 2006 American Chemical Society).

In the follow-on work, Choy *et al.* [50] obtained a similar MTX–LDH via a coprecipitation step and found that in an osteosarcoma cell culture line, the clathrin-mediated endocytosis of LDH nanoparticles also enhances the internalization of conjugated MTX molecules, and the MTX–LDH was more toxic to cancer cells than MTX alone due to the more effective penetration through the cell membrane (Figure 13). The anti-cancer efficiency of this MTX–LDH material was also investigated by *in vitro* bioassays such as 3-(4,5-dimethylthiazol-2-yl)-2,5-diphenyltetrazolium bromide (MTT) and BrdU (5-bromo-2-deoxyuridine) with the bone cancer cell culture lines (Sao-2 and MG-63) [86]. It was also found that the LDH host itself had no adverse effects on both normal and cancer cells up to a concentration of 500 μg·mL^−1^.

Thereafter, Choy *et al.* [87,88] further reported the structure of anti-cancer drug–LDH nanohybrid MTX–LDH and its cellular interaction with human breast adenocarcinoma MCF-7 cells and drug resistance upon enhanced permeability and retention effect of MTX–LDH nanoparticles.

**Figure 14:**
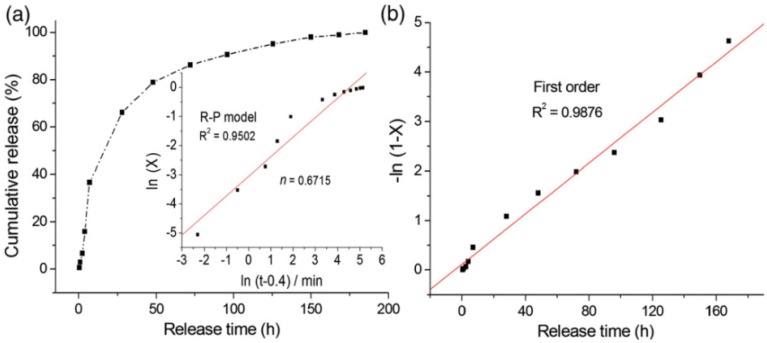
(**a**) Cumulative release of MTX from MgAl–LDH–MTX matrix as a function of time and (**b**) the release data fitted to the first order kinetics model. Reprinted with permission from [92] (Copyright 2013 Elsevier).

In the meantime, Chakraborty and Ghosh [88,89,90,91,92] also prepared MTX–LDH by different methods and studied the particle dissolution and subsequent drug release properties from the matrix in PBS solution. A striking enhancement in efficacy/sensitivity of MTX against HCT-116 cells was obtained when intercalated within the LDH host, as revealed by the attainment of the half maximal inhibitory concentration of MTX–LDH nanohybrid after only 48 h, whereas bare MTX required 72 h to achieve the same effect. The MTX release from MgAl–LDH–MTX hybrids in PBS at pH 7.4 followed relatively slow first order kinetics (Figure 14) and was complete within 8 days following diffusion and crystal dissolution mechanisms.

Chen *et al.* [93] developed a novel carrier for MTX by modifying an LDH host with folic acid (FA) molecules which had been covalently attached to (3-aminopropyl) triethoxysilane (APTES). The loading of MTX is 27.4 wt%. The FA-conjugated LDH nanoparticles greatly improve the efficiency of MTX and perform much better in killing cancer cells in comparison with free MTX.

5-Fluorouracil (5-Fu) is a neutral weak acid and anti-metabolic drug which is used extensively in cancer chemotherapy although it also has adverse effects on the human body. Wang *et al.* [94] first reported the intercalation of 5-Fu into LDHs in 2005 using the reconstruction method. The orientation of 5-Fu within the interlayer galleries of the LDH host can be varied by changing the aging treatment or the swelling agent. The basal spacings of the hybrids obtained at 60 °C and 70 °C were 1.06 and 0.8 nm, respectively, corresponding to a monolayer vertical and horizontal orientation to the layer, respectively, of the incorporated 5-Fu, whilst the hybrids obtained in glycerol with a basal spacing 1.24 nm may have a bilayer arrangement of 5-Fu anions. Different release mechanisms of 5-Fu were observed at varied pH values: an ion-exchange process between the 5-Fu anion pillared in the lamellar host and phosphate anions occurred in a buffer solution at pH 7 while ion-exchange and removal of the inorganic host due to the partial dissolution of the LDH host was observed at pH 4.

Choy *et al.* [95] also reported 5-Fu intercalated LDHs in 2008 using the coprecipitation method and evaluated the drug efficiency of 5-Fu–LDH against several cancer cell lines such as human lung cancer, osteosarcoma, and hepatoma cells and compared the results with those for MTX–LDH. Both 5-Fu–LDH and MTX–LDH nanohybrids more effectively inhibited cancer cell proliferation than free 5-Fu and MTX, respectively, though the performance of 5-FU–LDH was poorer than that of MTX–LDH.

**Figure 15:**
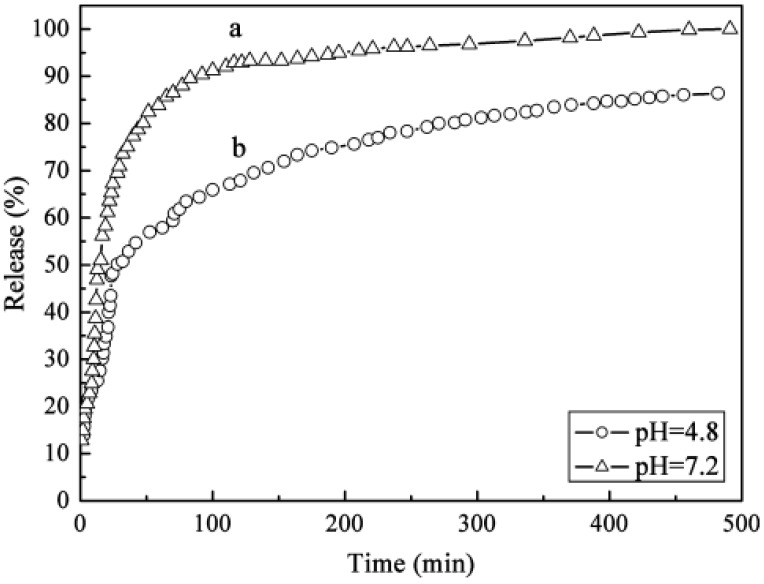
Release profiles of 5-Fu from 5-Fu/CMCD–LDH composite in phosphate-citrate buffer solution at different pH values. Reprinted with permission from [96] (Copyright 2010 American Chemical Society).

Wei *et al.* [96] studied the intercalation of an inclusion complex of 5-Fu and β-cyclodextrin (CMCD) into an LDH host using the ion-exchange step from a ZnAl–LDH–NO_3_ precursor. Spectroscopic studies showed that the structure of 5-Fu/CMCD inclusion complex is retained after immobilization in the LDH host. The release profiles of 5-Fu/CMCD in phosphate-citrate buffer (pH 4.8 and 7.2) at 37 °C (Figure 15) indicated that a faster release and higher release amount of 5-Fu were observed at pH 7.2 than in the acidic medium (pH 4.8). Inclusion of 5-Fu in the CMCD cavity prior to intercalation into LDH host prolongs the drug release time compared to the material obtained by direct intercalation of 5-Fu into the same LDH. The release of 5-Fu from drug/CMCD-LDH composite follows the Korsmeyer-Peppas equation very well at varied pH values. The rate determining step for release of 5-Fu from the 5-Fu/CMCD–LDH composite may be one of the following steps, depending on the conditions: (1) dissolution of LDH particles; (2) ion-exchange reaction between the inclusion complex 5-Fu/CMCD and citrate anions in the buffer solution; and (3) release of 5-FU from CMCD.

Xu *et al.* [97] reported another multifunctional nanovehicle for tumor optical imaging and therapy using Y_2_O_3_:Er^3+^,Yb^3+^ nanoparticles as near infrared fluorescent nanophosphors and MgAl–LDH–5-Fu nanosheets as drug nanovehicles. The hierarchically structured MgAl–LDH–5-Fu nanosheets were deposited on the surface of Y_2_O_3_:Er^3+^,Yb^3+^@SiO_2_ (via a urea assisted homogenous precipitation route) by a simple precipitation step followed hydrothermal treatment. The surface of the nanospheres consists of self-assembled 5-Fu–LDH layers with individual 5-Fu–LDH nanoplatelets standing vertically to form an open structure, with the thickness of the 5-Fu–LDH nanoplatelets being around 70–100 nm. The nanovehicle exhibits strong infrared upconversion fluorescence under excitation by a 980 nm laser which allows its extent of uptake by cancer cells to be monitored. The nanovehicle has a better anti-cancer efficiency than free 5-Fu attributed to its positively charged surfaces which results in a favorable interaction with the negatively charged cell membranes.

**Figure 16:**
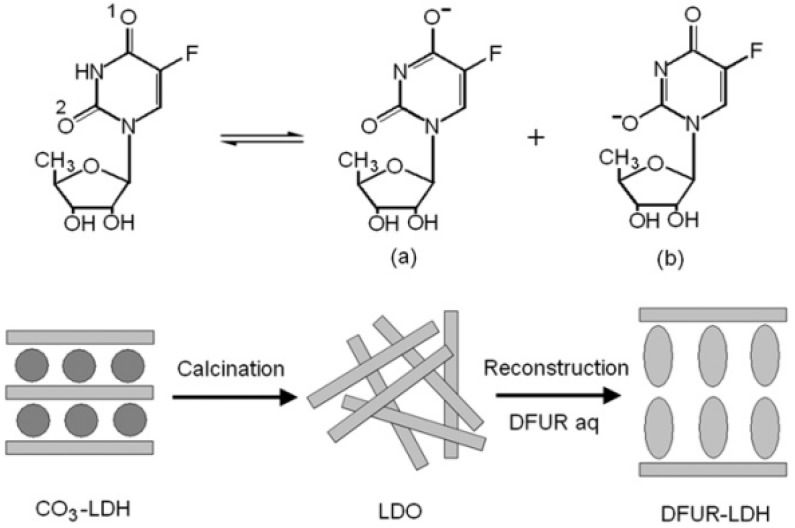
Resonance structure of doxifluridine (DFUR) and the synthetic strategy for DFUR–LDH hybrids. Modified with permission from [17] (Copyright 2010 Elsevier).

Doxifluridine (DFUR) is another important and frequently used anticancer drug with more effective and less toxic properties than 5-Fu. However, its application in clinical treatment is still limited by the side effects of oral administration such as diarrhea, nausea, and mucositis. Zhang *et al.* [17] fabricated a series of DUFR intercalated MgAl–LDH microhybrids with varied Mg/Al molar ratios (denoted as *r*) ranging from 1.7 to 2.9 and pH values (denoted as *p*) during aging step ranging from 7.2 to 10 via the reconstruction method (Figure 16).

**Figure 17:**
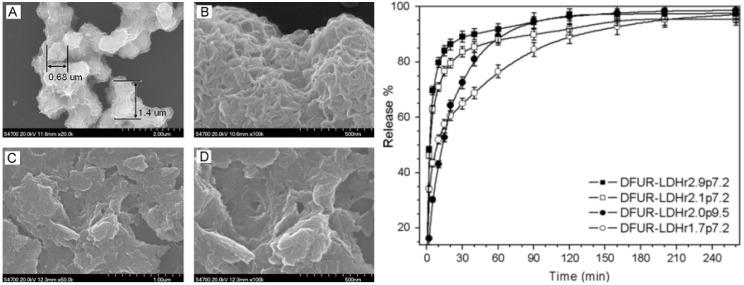
(**Left**) SEM images of DFUR–LDH*r*1.7*p*7.2 (**A**,**B**) and DFUR–LDH*r*2.0*p*9.5 (**C**,**D**); (**Right**) Release profiles of DFUR-LDH*rip*7.2 (*i* = 1.7, 2.1, 2.9) and DFUR–LDH*r*2.0*p*9.5. Modified with permission from [17] (Copyright 2010 Elsevier).

The arrangement of DFUR within the interlayer galleries of the LDH was found to depend on both Mg/Al ratios and the reaction pH values as a result of the different arrangements of the guest anions within the interlayer galleries, *i.e.*, with a bilayer interlayer arrangement for DFUR–LDH*r*1.7*p*7.2 and a more compact arrangement for DFUR–LDH*r*2.0*p*9.5. The particle morphologies also varied with both Mg/Al ratios and reaction pH values. For example, DFUR–LDH*r*1.7*p*7.2 consists of interconnected spheres with particle size of 500–700 nm and a worm-like morphology, which can be identified as the embryo of a rosette-like morphology, whilst DFUR–LDH*r*2.0*p*9.5 has much smaller irregular plate-like particles with observable breaks or holes (Figure 17(Left)).

The *in vitro* release profiles of DFUR from different DFUR–LDH microhybrids (Figure 17(Right)) showed that DFUR–LDH*r*2.0*p*9.5 gives a much faster release rate than DFUR–LDH*r*1.7*p*7.2, probably due to the discontinuous morphology and the much smaller particle size of the former. On the other hand, DFUR–LDH*r*2.1*p*7.2 and DFUR–LDH*r*2.9*p*7.2 present much faster release rates than DFUR–LDH*r*1.7*p*7.2, being attributable to their lower layer charge densities and poorer crystallinity. The release data fitted well with Bhaskar and modified Freundlich models, demonstrating a heterogeneous particle diffusion mechanism of DFUR from the DFUR–LDH microhybrids. Further proper modification by Eudragit^®^ L 100, the DFUR–LDH*r*1.7*p*7.2/L100 presents a readily controllable release behavior relied on the variation of the release medium pH values.

Some forms of active drugs are only slightly soluble in water, leading to poor dispersions in physiological solutions as well as difficulties in efficient dose delivery. In an attempt to overcome this problem, Giannelis *et al.* [98] first incorporated the non-ionic, poorly water-soluble camptothecin (CPT) into micelles derived from negatively charged surfactants, and then encapsulated in MgAl–LDH particles by an ion-exchange step. The LDH crystals have a typical hexagonal shape with dimensions below 500 nm in two directions with the third dimension corresponding to the stacked inorganic sheets approximately 10 nm. CPT was rapidly released from the resulting nanobiohybrids with complete release within 10 min at both pH 4.8 and 7.2. When the nanobiohybrid containing the CPT was administered to Glioma cells *in vitro*, their survival times were significantly lower compared to untreated cells, or to cells incubated with the surfactant, the pristine LDH, or water (the delivery medium).

**Figure 18:**
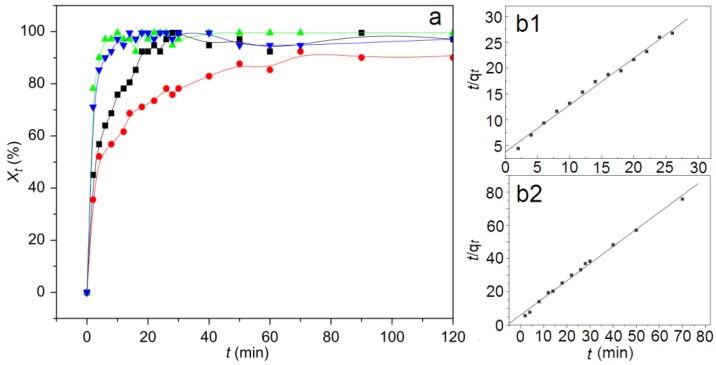
Release profiles (**a**) for CPT from a CPT–LDH composite and the physical mixture at pH 4.8 and pH 7.2 (■: composite, pH 4.8; ●: composite, pH 7.2; ▲: physical mixture, pH 4.8; ▼: physical mixture, pH 7.2) and linear regression curves of release data fitted with the pseudo-second-order kinetic model at pH 4.8 (**b1**) and pH 7.2 (**b2**) (*t* refers to release time, *X_t_* refers to percentage releases, *q_t_* refers to release amount at any time (*t*)). Modified with permission from [99] (Copyright 2010 Elsevier).

Hou *et al.* [99] prepared CPT–LDHs by a simple reconstruction of calcined LDHs using an organic solvent–water mixed solvent medium to increase the solubility of CPT. The composite is composed of very thin crimpled platelet-like particles of 100–200 nm. The CPT molecules are arranged as monolayer with their long axis parallel to the LDH layers. The *in vitro* drug release from the composites is significantly lower than that from the corresponding physical mixture. Hou *et al.* [100] recently reported the intercalation of CPT derivative 10-hydroxycamptothecin (HCPT) into sebacate-intercalated SC–LDHs upon hydrophobic interaction between HCPT and the parallel alkyl chains of monolayer arranged interlayer sebacate molecules. The obtained SC–HCPT–LDH composite consists of many nanosheets with a diameter of 300–500 nm and a thickness of 30–40 nm. The release kinetic studies (pH 7.2 PBS) (Figure 18) upon different kinetic models indicated that the pseudo-second-order model is a more satisfactory description for the HCPT release process from SC–HCPT–LDH nanocomposites, which is similar to the previous report for the CPT–LDH obtained by the reconstruction method [99].

The intercalation of other anti-cancer drugs in LDHs has been less widely discussed. For instance, a drug delivery system composed of Mg_2_Al–NO_3_ and K_7_[PTi_2_W_10_O_40_]·6H_2_O (PM-19) was designed by Wang *et al.* [101]. The Keggin-type heteropolyoxotungstate a-nion PM-19 shows high anti-viral and anti-tumor activity, but also has high toxicity resulting in adverse effects on the human body. The gallery height of the obtained Mg_2_Al–PM-19 is 0.99 ± 0.01 nm, in line with the spherical diameter of PM-19 anion. This material shows a non-uniform, irregularly agglomerated, compact, and non-porous plate-like structure. The observed rapid release rate and 100% release of PM-19 from the LDH composite after 60 min at pH 1 can be attributed to the complete collapse of the LDH layered structure. At pH 4 and pH 7, the rapid release during the first 60 min was followed by a more sustained release of the drug; this is because when the large PM-19 anions are replaced by smaller phosphate anions from the buffer solution, a phase boundary between the internal zone containing the intercalated PM-19 and the external zone with the phosphate anions leads to a decrease in the rate of drug release.

The same group also reported a nanohybrid podophyllotoxin–LDH (PPT–LDH) by a two-step method [102] giving a material with particles of 80–90 nm and a zeta potential of 20.3 mV. The PPT–LDH particles show better anti-tumor efficiency than free PPT and are more readily taken up by Hela cells. PPT–LDH shows a long-term suppression effect on tumor growth, and enhances the apoptotic process of tumor cells. A pharmacokinetics study showed that PPT–LDH had a prolonged circulation time and an increased bioavailability compared with free PPT.

**Figure 19:**
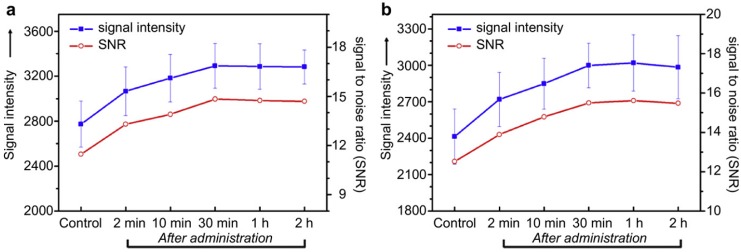
T_1_-weighted MRI signal intensity and signal to noise ratio (SNR) of liver (**a**) and spleen (**b**) before (control) and after intravenous administration of LDH–Gd/Au nanocomposite/physiological saline at various intervals (dose: 3.1 mg or 197.1 mmol·Gd/kg). Reprinted with permission from [103] (Copyright 2013 Elsevier).

Shi *et al.* [103] developed a Gd^3+^ ions (magnetic resonance imaging (MRI) contrast agent) doped LDH/Au (computed tomography (CT) contrast agent) nanocomposite as both a drug carrier and a diagnostic agent. The LDH–Gd/Au nanocomposite have a high surface area (112.9 m^2^·g^−1^), and thus high loading (264 mg (DOX)/g carrier) of the non-anionic anti-cancer drug doxorubicin by hydrogen bonding interaction, and the loaded DOX shows an interesting pH-responsive release, which is favorable for avoiding quick drug release in neutral blood system but promoting drug release at acidic tumor sites or within cells. Meanwhile, the nanocomposite has been found to be better CT and T_1_-weighted MRI contrast agents *in vitro* than commercial contrast agents, and effective CT and T_1_-weighted MRI contrast agents *in vivo* in reticuloendothelial systems such as liver and spleen and both CT and MR images present higher signal enhancements in spleen than in liver (Figure 19), suggesting that the synthesized disk-shaped LDH–Gd/Au nanocomposite is more easily enriched in spleen than in liver.

Based on above descriptions and discussions, it is concluded that the LDH nanoparticles can be considered as one of the ideal carriers for pharmaceutically active agents, and the obtained drug–LDH nanohybrid composites can be widely employed in future cardiovascular, anti-inflammatory and anti-cancer chemotherapies [104]. Besides the drugs mentioned above, some other kinds of drugs have also been co-assembled within LDH materials, including anti-histamine drugs [105], antioxidant drugs [106], peptide drugs [107], and amino acids [108]. Most of these drug–LDH composites show both good storage lifetimes because of the protection afforded by the LDH layers, and controlled release properties of the loaded drugs.

Table 1 summarizes all of the drug–LDH composites reviewed in this paper, indicating the intercalated drug, the chemical composition of the layers (metal cations), the preparation method, and a brief description of drug release conditions.

**Table 1:** Brief summary of LDH-based DDS reviewed in this paper.

Pharmaceutical guest	LDH host	Preparation	Drug Release	Ref.
Gemfibrozil	Li, Al	Ion-exchange	PBS (pH 4.0, 7.0)	[57]
Captopril	Mg, Al	Coprecipitation	PBS (pH 4.6, 7.4)	[58]
Heparin	Mg, Al	Coprecipitation	PBS (pH 7.4)	[59]
Pravastatin	Mg, Al	Coprecipitation	PBS (pH 7.5); HCl buffer (pH 4.5); simulated intestinal body fluids (pH 7.0)	[60]
Fluvastatin	Mg, Al	Coprecipitation	PBS (pH 7.5); HCl buffer (pH 4.5); simulated intestinal body fluids (pH 7.0)	[60]
Bezafibrate	Mg, Al	Coprecipitation	Simulated stomach sol. (pH 2.0); Simulated duodenum sol. (pH 6.0); Simulated intestine sol. (pH 8.0)	[61]
Clofibric acid	Mg, Al	Coprecipitation	Simulated stomach sol. (pH 2.0); Simulated duodenum sol. (pH 6.0); Simulated intestine sol. (pH 8.0)	[61]
Ibuprofen	Mg, Al	Ion-exchange	PBS (pH 7.5)	[63]
Ibuprofen	Mg, Al	Coprecipitation	HCl aqueous solution	[64]
Ibuprofen	Mg, Al	Ion-exchange	HCl aqueous solution	[64]
Ibuprofen	Mg, Al	Reconstruction	HCl aqueous solution	[64]
Cu(II)-ibuprofen	Mg, Al	Adsorption	HCl aqueous solution	[64]
Ibuprofen	Mg, Al	Ion-exchange	–	[65]
Ibuprofen	Mg, Al	Coprecipitation	PBS (pH 7.0)	[66]
Ibuprofen	Mg, Al	Reconstruction	PBS (pH 7.0)	[42]
Ibuprofen	Mg, Al	Hydrothermal	PBS (pH 7.45)	[43]
Ibuprofen	Zn, Al	Ion-exchange	PBS (pH 7.4)	[67]
Naproxen	Mg, Al	Ion-exchange	–	[68]
Naproxen	Mg, Al	Coprecipitation	PBS (pH 7.4)	[69,71]
Naproxen	Mg, Al	Reconstruction	–	[69]
Naproxen	Mg, Fe, Al	Coprecipitation	PBS (pH 7.5)	[70]
Naproxen	Mg, Fe, Al	Ion-exchange	PBS (pH 7.5)	[70]
Naproxen	Zn, Al	Ion-exchange	PBS (pH 6.86)	[72]
Diclofenac	Mg, Al	Ion-exchange	PBS (pH 6.8, 7.5)	[65,73,75]
Diclofenac	Zn, Al	Ion-exchange	Simulated intestinal fluid (pH: 7.5 ± 0.1); PBS (pH: 7.0 ± 0.1)	[74,75]
Diclofenac	Zn, Mg, Al	Ion-exchange	–	[75]
Diclofenac	Zn, Al	Coprecipitation	Physiological serum (pH 5.5)	[76]
Ketoprofen	Mg, Zn, Al	Ion-exchange	–	[75]
Ketoprofen	Zn, Al	Coprecipitation	Physiological serum (pH 5.5)	[76]
Chloramphenicol	Mg, Zn, Al	Ion-exchange	–	[75]
Chloramphenicol	Zn, Al	Coprecipitation	Physiological serum (pH 5.5)	[76]
Fenbufen	Mg, Al	Ion-exchange	PBS (pH 7.8)	[77]
Fenbufen	Li, Al	Ion-exchange	PBS (pH 7.8)	[77]
Fenbufen	Mg, Al	Coprecipitation	PBS (pH 6.8, 7.4, 7.8)	[78]
Fenbufen	Mg, Al, Fe	Coprecipitation	PBS (pH 7.5)	[79]
Fenbufen	Mg, Al, Fe	Ion-exchange	PBS (pH 7.5)	[79]
Fenbufen	Mg, Al, Fe	Reconstruction	PBS (pH 7.5)	[79]
Flurbiprofen	Mg, Al	Ion-exchange	PBS (pH 7.4)	[80]
Flurbiprofen	Zn, Al	Coprecipitation	PBS (pH 7.4)	[81]
Flurbiprofen	Mg, Al	Coprecipitation	–	[69]
Flurbiprofen	Mg, Al	Reconstruction	–	[69]
Indomethacin	Mg, Al	Coprecipitation	Swiss mice *in vivo*	[82]
Indomethacin	Mg, Al	Reconstruction	Swiss mice *in vivo*	[82]
Indomethacin	Mg, Al	Ion-exchange	–	[65]
Mefenamic acid	Mg, Fe, Al	Coprecipitation	Tris buffer (tris-hydroxymethyl-aminomethane + H_3_PO_4_ + lauryl sulphate, pH 9)	[70]
Mefenamic acid	Mg, Fe, Al	Ion-exchange	Tris buffer (tris-hydroxymethyl-aminomethane + H_3_PO_4_ + lauryl sulphate, pH 9)	[70]
Mefenamic acid	Mg, Al	Coprecipitation	–	[83]
Mefenamic acid	Mg, Al	Ion-exchange	–	[83]
Mefenamic acid	Mg, Al	Reconstruction	–	[83]
Meclofenamic acid	Mg, Fe, Al	Coprecipitation	PBS (pH 7.4)	[70]
Meclofenamic acid	Mg, Fe, Al	Ion-exchange	PBS (pH 7.4)	[70]
Meclofenamic acid	Mg, Al	Coprecipitation	–	[83]
Meclofenamic acid	Mg, Al	Ion-exchange	–	[83]
Meclofenamic acid	Mg, Al	Reconstruction	–	[83]
5-Aminosalicylate	Zn, Al	Coprecipitation	–	[30]
5-Aminosalicylate	Zn, Al	Ion-exchange	–	[30]
Dexamethasone	Mg, Al	Coprecipitation	PBS (pH 7.4)	[84]
Folinic acid	Mg, Al	Ion-exchange	Human tendinous fibroblast cell line and human osteosarcoma cell line (SaOS-2) *in vitro*	[48]
Methotrexate	Mg, Al	Ion-exchange	Human tendinous fibroblast cell line and human osteosarcoma cell line (SaOS-2) *in vitro*	[48]
Methotrexate	Mg, Al	Coprecipitation	MNNG/HOS cells *in vitro*	[50]
Methotrexate	Mg, Al	Coprecipitation	Human osteosarcoma cell lines; Saos-2 & MG-63	[86]
Methotrexate	Mg, Al	Coprecipitation	Deionized water; Hank’s balanced salt solution; human breast adenocarcinoma MCF-7 cells	[87]
Methotrexate	Mg, Al	Coprecipitation	Parental HOS and MTX-resistant HOS/Mtx cells	[88]
Methotrexate	Mg, Al	*Ex situ* ion-exchange	PBS (pH 7.4)	[89]
Methotrexate	Mg, Al	*In situ* coprecipitation	PBS (pH 7.4)	[89]
Methotrexate	Mg, Al	*Ex situ* coprecipitation	PBS (pH 7.4)	[89]
Methotrexate	Zn, Al	Ion-exchange	PBS (pH 7.4)	[90]
Methotrexate	Mg, Al	Ion-exchange	PBS (pH 7.4)	[91]
Methotrexate	Mg, Al	*In situ* ion-exchange	PBS (pH 7.4)	[92]
Methotrexate	Mg, Al	Coprecipitation	Human lung cancer; osteosarcoma; hepatoma cells	[95]
5-Fuorouracil	Mg, Al	Reconstruction	PBS (pH 4, 7)	[94]
5-Fuorouracil	Mg, Al	Coprecipitation	Human lung cancer; osteosarcoma; hepatoma cells	[95]
5-Fuorouracil	Zn, Al	Ion-exchange	PBS (pH 4.8, 7.2)	[96]
5-Fuorouracil	Mg, Al	Precipitation & hydrothermal	MCF-7 cells	[97]
Doxifluridine	Mg, Al	Reconstruction	PBS (pH 7.45)	[17]
Camptothecin	Mg, Al	Ion-exchange	Glioma cells *in vitro*	[98]
Camptothecin	Mg, Al	Reconstruction	PBS (pH 4.8, 7.2)	[99]
10-Hydroxy-camptothecin	Zn, Al	Hydrophobic interaction	0.1 M PBS (pH 7.2)	[100]
K_7_[PTi_2_W_10_O_40_]·6H_2_O	Mg, Al	Ion-exchange	PBS (pH 1, 4, 7)	[101]
Podophyllotoxin	Mg, Al	Ion-exchange	Healthy female nude mice	[101]
Doxorubicin	Mg, Al	Hydrogen bonding interaction	L929 cells and HeLa cells *in vitro*; Kunming mice	[103]
Cetirizine	Mg, Al	Ion-exchange	PBS (pH 4.8, 7.4)	[105]
Cetirizine	Zn, Al	Ion-exchange	PBS (pH 4.8, 7.4)	[105]
Carnosine	Mg, Al	Ion-exchange	PBS (pH 7.4)	[106]
Gallic acid	Mg, Al	Coprecipitation	PBS (pH 7.4)	[106]
Glycy-l-Tyrosine	Mg, Al	Solvent evaporation	PBS (pH 7.4)	[107]
l-Tyrosine	Mg(Ni, Zn), Al	Coprecipitation	–	[108]

As far as we can see, LDHs material has been successfully combined with pharmaceutical drug molecules and shows great potential for drug delivery. However, the crucial challenges in efficiently delivering drug guests by LDH carrier still exist. One is the inevitable or still not clearly revealed chemical and biological toxicity of these drug–LDH nanoparticles despite their generally lower level compared with other inorganic nanoparticles. Another one is the non-selectivity of the drug–LDH for the targeted sites, resulting in an aimless and less efficient delivery. The third one is the less precise control of particle size and size distribution which may limit the efficient transportation of drug–LDH *in vivo* considering the possible damage of tissue or the phagocytosis of phagocytic cells. All these issues may alternatively rely on the modification or functionalization of drug–LDH hybrid which can promote the biological affinity to organism, the recognition capability to lesions, and the ability of delivery *in vivo*.

For the most commonly studied cardiovascular drugs and anti-inflammatory drugs mentioned above, the aim of sustained and controlled release has already been achieved practically. However, for the same kinds of drugs like anti-cancer drugs which need special requirements of release process with precise control to targeted site, further studies are still needed. Hence, novel structures and synthetic strategies are continuously being proposed [40,109,110,111] to solve this problem.

The introduction of a third component such as magnetic materials greatly pushes the functionalization process forward. Magnetic nanoparticles exhibit unique properties such as superparamagnetism [112], remote manipulation, magnetic imaging, and hyperthermic effect [113]. The use of magnetic nanoparticles affords greater flexibility to adjust the release process to improve the availability of drug molecules to the targeting therapeutic sites, and magnetic nanoparticles hybridized with drug–LDHs [114,115,116,117] are becoming one class of novel hierarchically structured bio-nanohybrid materials showing great application potential, which will be reviewed in the future.

## 3. Conclusions

By taking advantage of their ease of synthesis, structural diversity and stability, numerous LDH-based drug carrier systems have been employed as drug delivery system (DDS) and shown potential practical applications for the controlled release of a variety of pharmaceutical active agents. Most published studies have focused on the investigation of drug–LDH interactions and the *in vitro* behavior of drug guests in depth. The therapeutic advantages of LDH-based drug carriers make this field of investigation a high-priority development area. Nevertheless, some challenging problems still remain, particularly in respect to the synthesis of hierarchical complex structures with multifunctionality and the targeting of high efficiency release *in vivo*, and further results in these areas are eagerly awaited.
